# Impact of the metabolic syndrome on severe mental disorders

**DOI:** 10.1007/s00406-020-01156-5

**Published:** 2020-06-26

**Authors:** Andrea Schmitt, Daniela Reich-Erkelenz, Peter Falkai

**Affiliations:** 1 Department of Psychiatry and Psychotherapy, University Hospital, LMU Munich, Nußbaumstrasse 7, 80336 Munich, Germany; 2 Institute of Psychiatric Phenomics and Genomics, University Hospital, LMU Munich, Nußbaumstrasse 7, 80336 Munich, Germany

Compared to the general population, severe psychiatric disorders are associated with an increased mortality, which can largely be explained by a high incidence of somatic comorbidities, unhealthy lifestyle, low physical activity and increased rate of suicides [1]. For example, schizophrenia patients have a higher prevalence of the metabolic syndrome than the general population, with a two- to threefold increased risk for cardiovascular diseases (CVDs) resulting in increased cardiac mortality [2]. This leads to an estimated reduction in life expectancy of nearly 10–15 years compared to the general population. The metabolic syndrome is defined as a combination of increased waist circumference and two of the following criteria: high blood pressure, elevated triglycerides, low high-density lipoprotein (HDL) cholesterol and elevated fasting glucose, by the International Diabetes Federation. It is also defined by the Adult Treatment Panel III (ATP III) of the National Cholesterol Education and the adapted Adult Treatment Panel III (ATP III-A) of the American Heart Association, which require 3 criteria to be fulfilled [1].

The high prevalence of the metabolic syndrome has an impact on clinical outcome in patients with severe mental disorders. Using the IDF and ATP III criteria, 30.1% of psychiatric inpatients were found to be overweight, 17.2% obese and about 25% fulfilled criteria for metabolic syndrome. In fact, 3.8% had (pre)diabetes, 8.3% had a moderate and 1.9% a high CVD score [3]. In a follow-up study of schizophrenia patients, prevalence of the metabolic syndrome increased substantially after a mean of 8 years and the nutritional status was found to play a major role [4]. In patients with psychosis, high cholesterol and low-density lipoprotein (LDL) scores showed significant associations with depression [5]; while, an increase in HDL levels was related to improvement in verbal learning [6]. In trauma-exposed women, higher body mass index was associated with less cardiac output and heart rate recovery from a stress task, suggesting that PTSD recovery may be mediated by body mass index [7].

A position statement from the European Psychiatric Association (EPA) recommends the use of physical activity in routine clinical care of severe mental disorders such as schizophrenia and major depression to improve psychiatric and medical outcomes [8]. Aerobic exercise is affecting multiple organs and may positively influence neuroplastic processes as well as factors of the metabolic syndrome [9]. Therefore, current guidelines such as the German S3 Schizophrenia Guideline recommend its application in severe mental disorders. Further research is required on the neurobiological mechanisms by which aerobic exercise exerts its protective effects on factors of the metabolic syndrome and cognition. Furthermore, it should be investigated which mode and what duration of training are expected to have beneficial effects on cardiorespiratory fitness, metabolic parameters and symptoms of severe mental disorders [10].

## References

[1] Schmitt A, Maurus I, Rossner MJ, Röh A, Lembeck M, von Wilmsdorff M, Takahashi S, Rauchmann B, Keeser D, Hasan A, Malchow B, Falkai P (2018). Effects of aerobic exercise on metabolic syndrome, cardiorespiratory fitness, and symptoms in schizophrenia include decreased mortality. Front Psychiatry.

[2] Ringen PA, Engh JA, Birkenaes AB, Dieset I, Andreassen OA (2014). Increased mortality in schizophrenia due to cardiovascular disease—a non-systematic review of epidemiology, possible causes, and interventions. Front Psychiatry.

[3] Barton BB, Zagler A, Engl K, Rihs L, Musil R (2020). Prevalence of obesity, metabolic syndrome, diabetes and risk of cardiovascular disease in a psychiatric inpatient sample: results of the Metabolism in Psychiatry (MiP) Study. Eur Arch Psychiatry Clin Neurosci.

[4] Yoca G, Yağcıoğlu AEA, Eni N, Karahan S, Türkoğlu I, Yıldız EA, Mercanlıgil SM, Yazıcı MK (2020). A follow-up study of metabolic syndrome in schizophrenia. Eur Arch Psychiatry Clin Neurosci.

[5] Gohar SM, Dieset I, Steen NE, Mørch RH, Iversen TS, Steen VM, Andreassen OA, Melle I (2019). Association between serum lipid levels, osteoprotegerin and depressive symptomatology in psychotic disorders. Eur Arch Psychiatry Clin Neurosci.

[6] Gjerde PB, Simonsen CE, Lagerberg TV, Steen NE, Ueland T, Andreassen OA, Steen VM, Melle I (2020). Improvement in verbal learning over the first year of antipsychotic treatment is associated with serum hdl levels in a cohort of first episode psychosis patients. Eur Arch Psychiatry Clin Neurosci.

[7] Kibler JL, Ma M, Llabre MM (2020). Body mass index in relation to cardiovascular recovery from psychological stress among trauma-exposed women. Eur Arch Psychiatry Clin Neurosci.

[8] Stubbs B, Vancampfort D, Hallgren M, Firth J, Brand S, Cordes J, Malchow B, Gerber M, Schmitt A, Correll C, DeHert M, Gaughran F, Schneider F, Veronese N, Solmi M, Falkai P, Möller H-J, Kahl KG (2018). EPA guidance on physical activity as a treatment for severe mental illness: a meta-review of the evidence and Position Statement from the European Psychiatric Association (EPA), supported by the International Organization of Physical Therapists in Mental Health (IOPTMH). Eur Psychiatry.

[9] Maurus I, Hasan A, Röh A, Takahashi S, Rauchmann B, Keeser D, Malchow B, Schmitt A, Falkai P (2019). Neurobiological effects of aerobic exercise, with a focus on patients with schizophrenia. Eur Arch Psychiatry Clin Neurosci.

[10] Schmitt A, Reich-Erkelenz D, Hasan A, Falkai P (2019). Aerobic exercise in mental disorders: from basic mechanisms to treatment recommendations. Eur Arch Psychiatry Clin Neurosci.

